# The efficiency of intravenous acetaminophen for pain control following total knee and hip arthroplasty: A systematic review and meta-analysis

**DOI:** 10.1097/MD.0000000000008586

**Published:** 2017-11-17

**Authors:** Limin Liang, Ying Cai, Aixiang Li, Chuangen Ma

**Affiliations:** Department of Anesthesiology, Huaihe Hospital, Henan University, P.R. China.

**Keywords:** acetaminophen, pain control, total hip arthroplasty meta-analysis, total knee arthroplasty

## Abstract

**Background:**

This meta-analysis aimed to evaluate the efficiency and safety of intravenous acetaminophen as an adjunct to multimodal analgesia for pain control after total joint arthroplasty (TJA).

**Methods:**

PubMed, Embase, Web of science, Medline, and Cochrane library databases were systematically searched. Randomized controlled trials (RCTs) and non-RCTs were included. Fixed/random effect model was used according to the heterogeneity tested by *I*^2^ statistic. Meta-analysis was performed using Stata 11.0 software.

**Results:**

Four studies including 865 patients met the inclusion criteria. The present meta-analysis indicated that there were significant differences between groups in terms of pain scores at 24 hours (weighted mean difference [WMD] = −0.926, 95% confidence interval [CI]: −1.171 to −0.681, *P* = .000), 48 hours (WMD = −0.905, 95% CI: −1.198 to −0.612, *P* = .000), and 72 hours (WMD = −0.279, 95% CI: −0.538 to −0.021, *P* = .034). Significant differences were found regarding opioid consumption at 24 hours (WMD = −4.043, 95% CI: −5.041 to −3.046, *P* = .000), 48 hours (WMD = −5.665, 95% CI: −7.383 to −3.947, *P* = .000), and 72 hours (WMD = −6.338, 95% CI: −7.477 to −5.199, *P* = .000).

**Conclusion:**

Intravenous acetaminophen was efficacious for reducing postoperative pain and opioid consumption than the placebo following total joint arthroplasty. Due to the limited quality of the evidence currently available, more RCTs are needed.

## 1 Introduction

Total joint arthroplasty (TJA) is popular surgical procedures for improving quality of life and functional outcome for patients with end-stage osteoarthritis.^[1–3]^ Severe pain after TJA may cause discomfort and stress which is an important clinical challenge.^[4]^ Inadequate pain management following TJA is associated with poor postoperative rehabilitation and a prolonged length of hospital stay. Pain management following TJA remains an interesting topic and the optimal methods remain controversial.

Various analgesics are prescribed for perioperative pain including nerve block, intravenous anesthetics and local infiltration anesthesia (LIA).^[5–8]^ However, these methods are unable to provide sufficient analgesia. Thus, additional opioid was commonly used. Adverse effects including gastrointestinal events, headache, and urinary retention limited the clinical application. The American Society of Anesthesiologists (ASA) has highly recommend that multimodal analgesia was an effective method for pain control after major orthopedic surgery.

Recently, acetaminophen is used as analgesic which is widely recognized for pain management in surgical procedures.^[9]^ Previous systematic review has demonstrated that use of acetaminophen was efficacious in reducing pain and morphine consumption in patients with cancer. The mechanism of acetaminophen is to selectively inhibit Cycloxygenase (COX) activities, which can play a role in treating fever and pain. It does not block an active site directly, but rather by decreasing COX, which should be oxidized in order to function.^[10]^

Currently, there is lack of reliable evidence for the use of acetaminophen as an adjunct to a multimodal analgesia regimen for pain management following TJA. Thus, we conduct a meta-analysis from recent published articles to evaluate the efficacy of acetaminophen for pain control following total knee and hip arthroplasty.

## 2 Methods

This meta-analysis was performed in accordance with the preferred reporting items for systematic reviews and meta-analyses (PRISMA) guidelines. All analyses were based on previous studies, therefore, no ethical approval are required.

### 2.1 Search strategy

**Figure 1:**
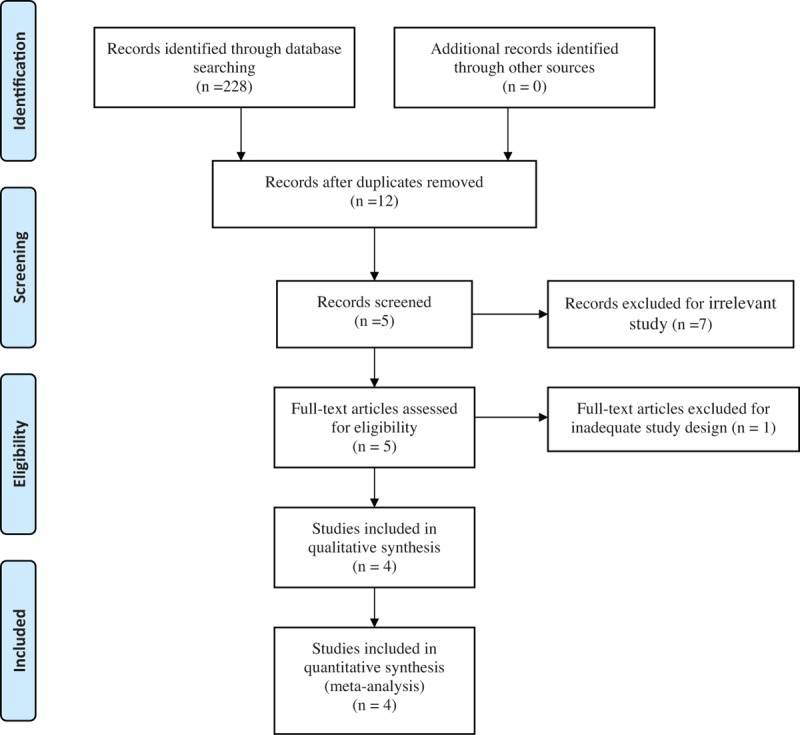
Search results and the selection procedure.

Two researchers search the relevant studies independently including Embase (1980–2017.09), PubMed (1966–2017.09), ScienceDirect (1985–2017.09), Web of Science (1950–2017.09), and Cochrane Library for potential relevant studies. Reference lists of all the potential included studies and relevant reviews were hand-searched for any additional trials. No restrictions were imposed on language. The Mesh terms and their combinations used in the search were as follows: “total knee replacement OR arthroplasty,” “total hip replacement OR arthroplasty,” “acetaminophen,” and “pain management.” The retrieval process is presented in Fig. 1. A third reviewer acted as a judge if there was any disagreement.

### 2.2 Inclusion criteria

Studies were considered eligible if they met the following criteria: published clinical randomized controlled trials (RCTs) and non-RCTs; patients undergoing total joint arthroplasty, experiment group received intravenous acetaminophen for pain control and control group received normal saline or none; the primary outcomes included postoperative pain scores and morphine equivalent consumption. Secondary outcomes included length of hospital stay and postoperative adverse effects such as nausea and vomiting. Studies would be excluded from current meta-analysis for incomplete data, case reports, conference abstract, or review articles.

### 2.3 Study selection

Two authors independently reviewed all the abstracts of the potential studies identified by the above searches. After an initial decision, full text of the studies that potentially met the inclusion criteria were reviewed and final decision was made. A senior reviewer is consult in case of disagreement regarding which studies to include.

### 2.4 Date extraction

Two authors independently extracted the relevant data from the included articles. Details of incomplete data of included studies are obtained by consulting the corresponding author. Following data were extracted: first author names, published year, sample size, study design, comparable baseline, analgesic methods, and duration of follow-up. Other relevant data were also extracted from individual studies.

### 2.5 Assessment of methodological quality

Quality assessment of the included RCTs was assessed by 2 authors independently which used the Cochrane Collaboration's tool. We conducted “risk of bias” table including the following key points: random sequence generation, allocation concealment, blinding, incomplete outcome data, free of selective reporting, and other bias, each item was recorded by “Yes,” “No,” or “Unclear.” Each risk of bias item was presented as a percentage across all included studies. The percentage indicated the proportion of different levels of risk of bias for each item. For non-RCTs, methodological Index for Non-Randomized Studies (MINORS) scale was applied to evaluate the methodological quality, which was based on the 12 main items.

The qualities of evidence of main outcomes were assessed using the Grading of Recommendations Assessment, Development and Evaluation (GRADE) system including the following items: risk of bias, inconsistency, indirectness, imprecision, and publication bias.^[11]^ Two authors independently score all the items of the GRADE systems which may influence quality of evidence. Items that may raise the quality of evidence was recorded by 0, +1, and +2. Items that may lower the quality of evidence was recorded by 0, −1, and −2. A senior reviewer is consult in case of disagreement. Finally, GRADE systems will overall evaluate the results. The recommendation level of evidence is classified into the following categories: high, which means that further research is unlikely to change confidence in the effect estimate; moderate, which means that further research is likely to significantly change confidence in the effect estimate and may change the estimate; low, which means that further research is likely to significantly change confidence in the effect estimate and to change the estimate; and very low, which means that any effect estimate is uncertain.

### 2.6 Data analysis and statistical methods

All calculations were carried out with Stata 11.0 (The Cochrane Collaboration, Oxford, United Kingdom). Statistical heterogeneity was assessed based on the value of *P* and *I*^2^ using the standard chi-squared test. When *I*^2^ > 50%, *P* < .1 was considered to be significant heterogeneous. The random-effect model was performed for meta-analysis; otherwise, the fixed-effect model was used. The results of dichotomous outcomes were expressed as risk difference (RD) with 95% confidence intervals (CIs). For continuous various outcomes, weighted mean difference (WMD) with a 95% CIs was applied for assessment.

## 3 Results

### 3.1 Search result

In the primary search, 228 articles were reviewed. Finally, 3 RCTs^[12–14]^ and 1 non-RCTs^[15]^ met eligibility criteria of the present meta-analysis. Overall, the 4 studies included 534 patients in the acetaminophen groups and 331 patients in the control groups.

### 3.2 Study characteristics

Demographic characteristics of the included studies are summarized in Table 1. The sample size of the included studies ranged from 66 to 609. All of them evaluated the efficiency of acetaminophen for pain management in TJA. Experiment group received intravenous acetaminophen for pain management and control group received normal saline or none. The follow-up period ranged from 1 to 12 months.

**Table 1:**
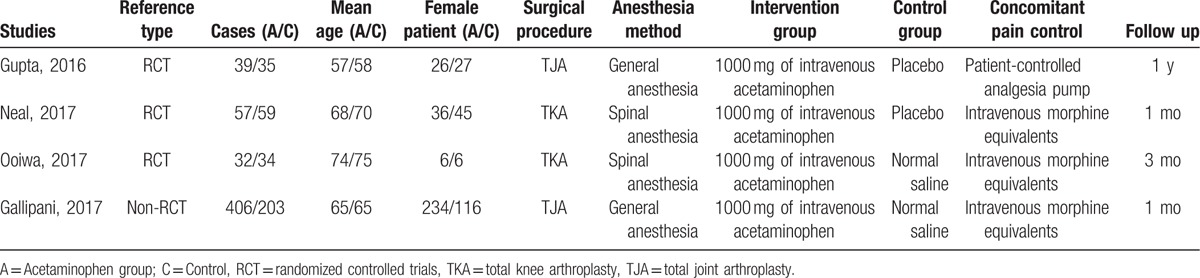
Trials characteristics.

### 3.3 Risk of bias

Quality assessment of the RCTs was based on the Cochrane Collaboration's tool (Table 2). Clear inclusion and exclusion criteria were described in all studies^[12–14]^ and all of them reported that eligible participants were randomized with a computerized random number generator. Two articles^[12,13]^ provided that sealed envelope was selected to make sure allocate concealment. All included articles confirmed double blinding. Each risk of bias item is shown as the percentage across all included RCTs (Table 3). The quality assessment for non-RCT was shown in Table 4.

**Table 2:**
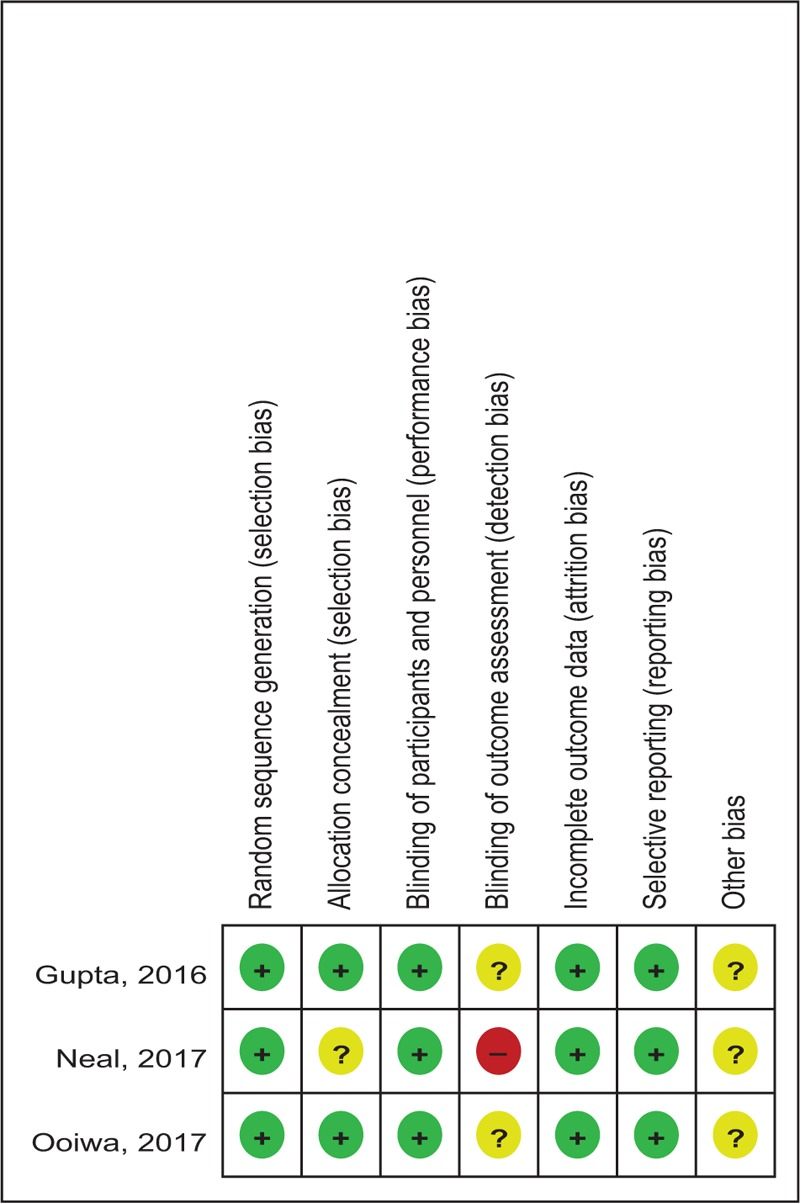
Methodological quality of the randomized controlled trials.

**Table 3:**
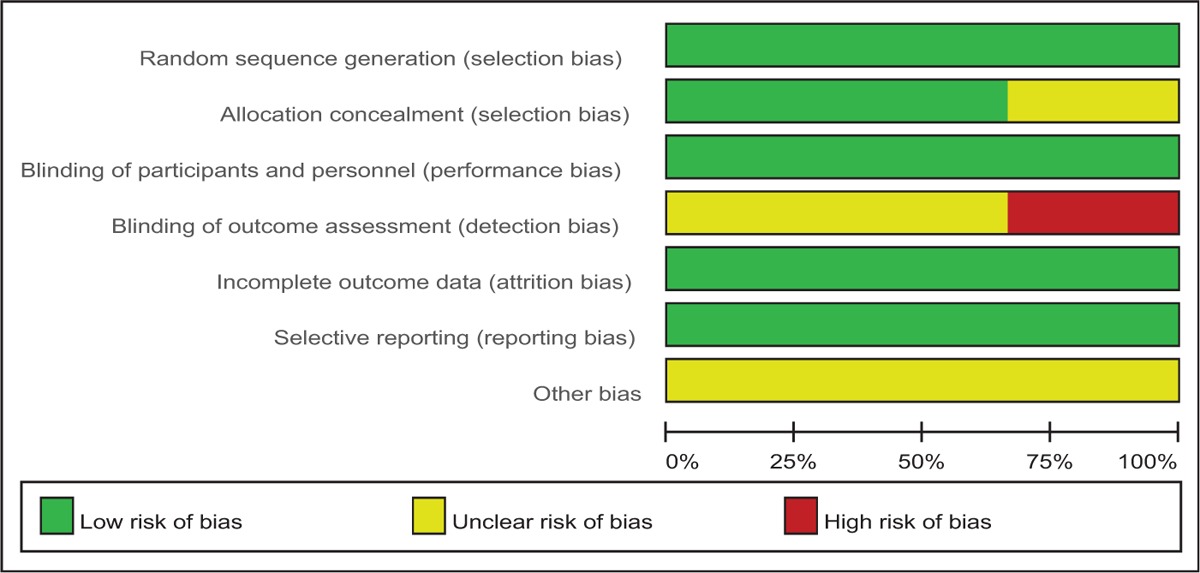
Risk of bias.

**Table 4:**
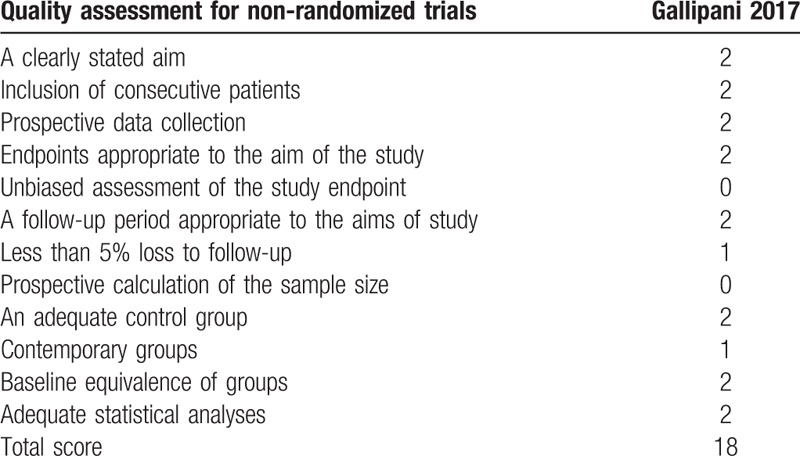
Methodological quality of the non-randomized controlled trials.

### 3.4 Outcomes for meta-analysis

#### 3.4.1 Pain scores at 24 hours

**Figure 2:**
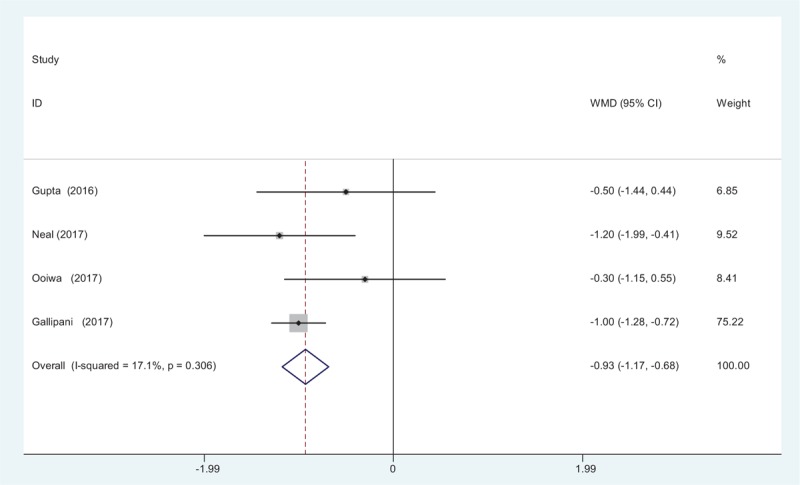
Forest plot diagram showing pain scores at 24 hours after TJA. TJA = total joint arthroplasty.

Four articles^[12–15]^ showed the pain scores at 24 hours following TJA. A fixed-effects model was adopted because no significant heterogeneity was found among the articles (*χ*^2^ = 3.62, *df* = 3, *I*^2^ = 0%, *P* = .306). The pooled results indicated that there was significant difference between groups regarding the pain scores at 24 hours (WMD = −0.926, 95% CI: −1.171 to −0.681, *P* = .000; Fig. 2).

#### 3.4.2 Pain scores at 48 hours

**Figure 3:**
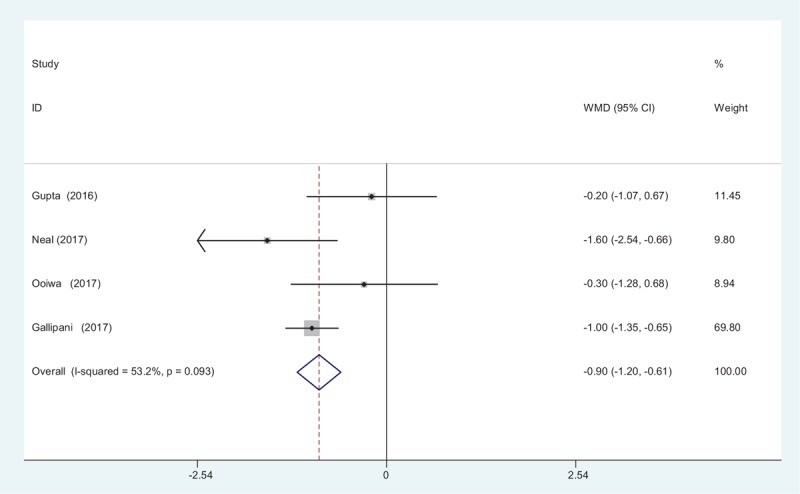
Forest plot diagram showing pain scores at 48 hours after TJA. TJA = total joint arthroplasty.

Four articles^[12–15]^ reported the outcome of pain scores at 48 hours following TJA. A fixed-effects model was used because no significant heterogeneity was found among the studies (*χ*^2^ = 6.41, *df* = 3, *I*^2^ = 53.2%, *P* = .093). There was significant difference in pain scores at 48 hours between groups (WMD = −0.905, 95% CI: −1.198 to −0.612, *P* = .000; Fig. 3).

#### 3.4.3 Pain scores at 72 hours

**Figure 4:**
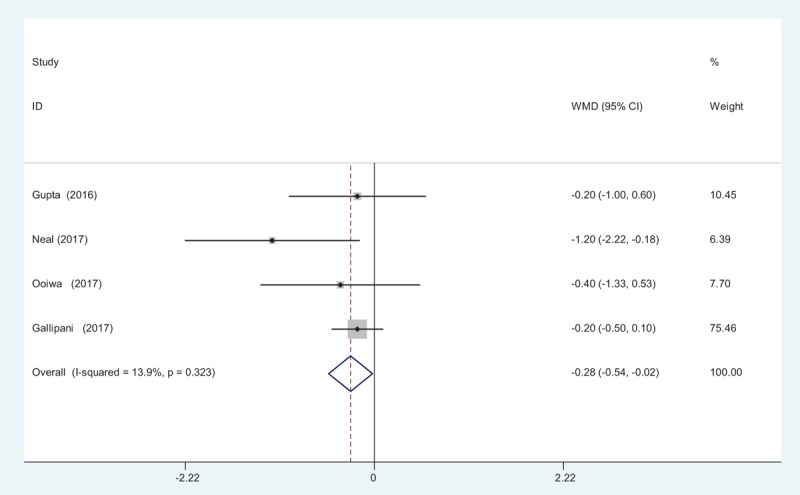
Forest plot diagram showing pain scores at 72 hours after TJA. TJA = total joint arthroplasty.

Four studies^[12–15]^ reported the outcome of pain scores at 72 hours following TJA. A fixed-effects model was used because no significant heterogeneity was found among the studies (*χ*^2^ = 3.49, *df* = 3, *I*^2^ = 13.9%, *P* = .323). The pooled results demonstrated that significant difference in VAS scores at 72 hours was identified between groups (WMD = −0.279, 95% CI: −0.538 to −0.021, *P* = .034; Fig. 4).

#### 3.4.4 Opioid consumption at 24 hours

**Figure 5:**
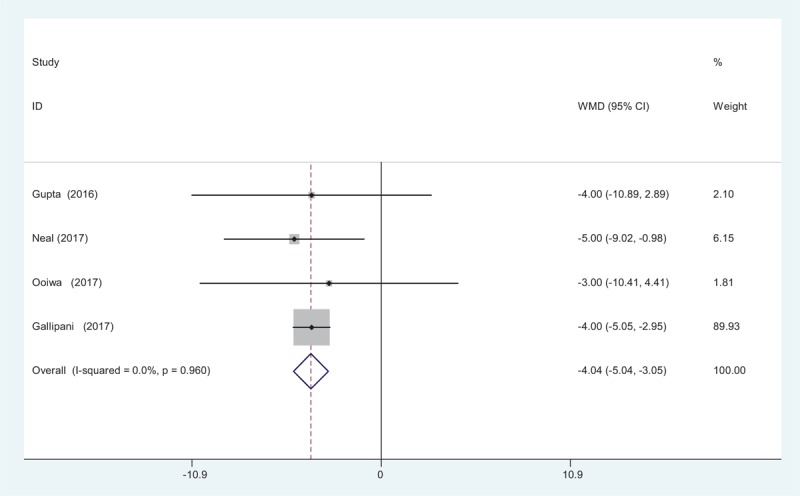
Forest plot diagram showing opioid consumption at 24 hours after TJA. TJA = total joint arthroplasty.

Intravenous patient-controlled analgesia (PCA) morphine consumption at 24 hours was reported in 4 studies.^[12–15]^ No significant heterogeneity was found among these studies (*χ*^2^ = 0.30, *df* = 3, *I*^2^ = 0%, *P* = .960) and a fixed-effects model was used. Significant difference was detected in opioids consumption at 24 hours between the 2 groups (WMD = −4.043, 95% CI: −5.041 to −3.046, *P* = .000; Fig. 5).

#### 3.4.5 Opioid consumption at 48 hours

**Figure 6:**
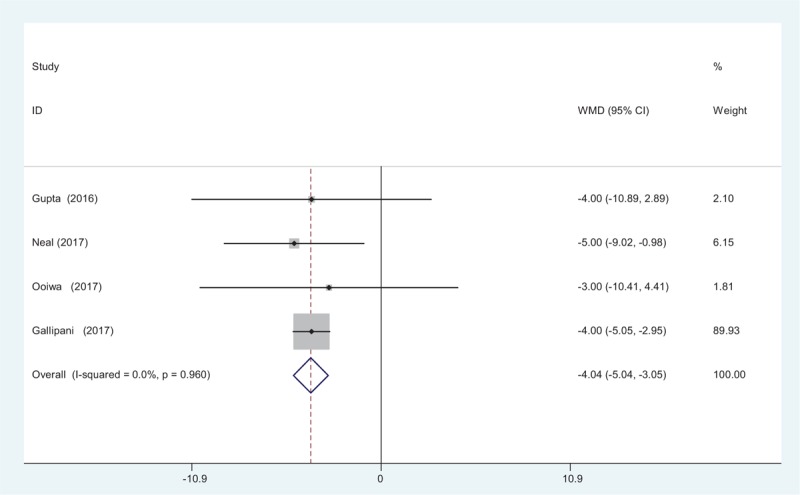
Forest plot diagram showing opioid consumption at 48 hours after TJA. TJA = total joint arthroplasty.

Four articles^[12–15]^ provided the outcome of intravenous PCA morphine consumption at 48 hours following TJA. A fixed-effects model was adopted because no significant heterogeneity was found (*χ*^2^ = 0.62, *df* = 3, *I*^2^ = 0%, *P* = .892). There was significant difference in opioid consumption at 48 hours between groups (WMD = −5.665, 95% CI: −7.383 to −3.947, *P* = .000; Fig. 6).

#### 3.4.6 Opioid consumption at 72 hours

**Figure 7:**
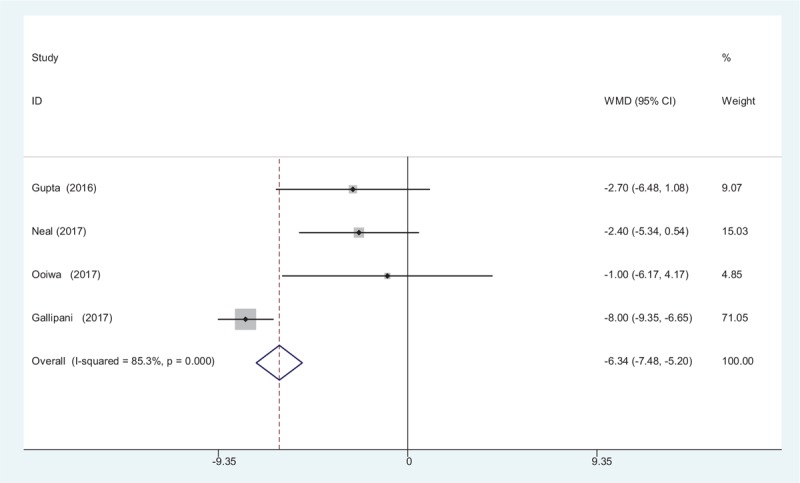
Forest plot diagram showing opioid consumption at 72 hours after TJA. TJA = total joint arthroplasty.

Four studies^[12–15]^ reported the outcomes of intravenous PCA morphine consumption at 72 hours following TJA. A random-effects model was applied (*χ*^2^ = 20.36, *df* = 3, *I*^2^ = 85.3%, *P* = .000). Significance difference in opioids consumption at 72 hours was observed between the 2 groups. (WMD = −6.338, 95% CI: −7.477 to −5.199, *P* = .000; Fig. 7).

#### 3.4.7 Length of hospital stay (LOS)

**Figure 8:**
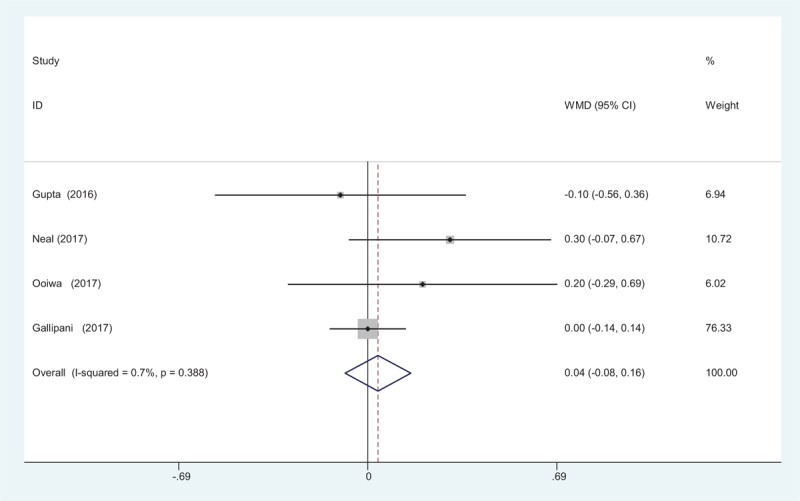
Forest plot diagram showing length of hospital stay after TJA. TJA = total joint arthroplasty.

Length of hospital stay was reported in 4 studies.^[12–15]^ No significant difference in the LOS was observed between the 2 groups (WMD = 0.037, 95% CI: −0.083 to 0.157, *P* = .544; Fig. 8).

#### 3.4.8 Nausea and vomiting

**Figure 9:**
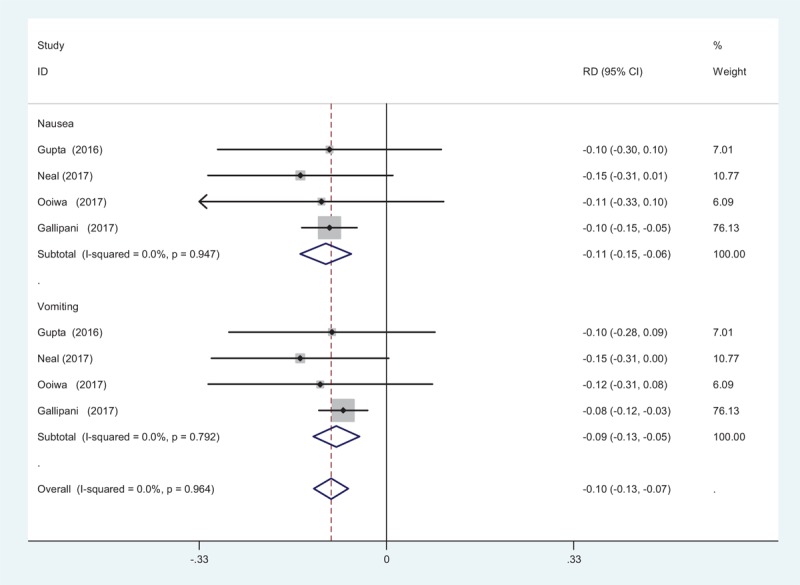
Forest plot diagram showing incidence of nausea and vomiting after TJA. TJA = total joint arthroplasty.

Four studies^[12–15]^ reported the postoperative complications of nausea following TJA. A fixed-effects model was adopted because no significant heterogeneity was found among the articles (*χ*^2^ = 0.36, *df* = 3, *I*^2^ = 0%, *P* = .947). Significant difference in the incidence of nausea was found between the 2 groups (RD = −0.107, 95% CI: −0.152 to −0.062, *P* = 0.000; Fig. 9). Four articles^[12–15]^ showed the postoperative complications of vomiting following TJA. A fixed-effects model was used (*χ*^2^ = 1.04, *df* = 3, *I*^2^ = 0%, *P* = .792). The pooled results demonstrated that there was an increased risk of vomiting in control groups (RD = −0.082, 95% CI: −0.123 to −0.042, *P* = .000; Fig. 9).

### 3.5 Evidence level

All main outcomes in this meta-analysis were evaluated using the GRADE system (Table 5).

**Table 5:**
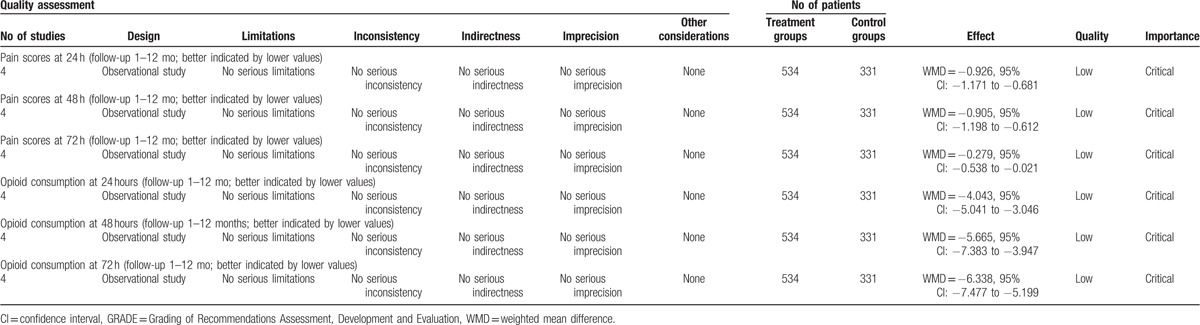
The GRADE evidence quality for main outcome.

The overall evidence quality for each outcome was moderate to low which means that further research is likely to significantly change confidence in the effect estimate and to change the estimate. This finding may lower the confidence in any recommendations.

## 4 Discussion

This meta-analysis aimed to review the related articles to obtain a better understanding of the effectiveness of intravenous acetaminophen for pain management following TJA. The result of the present meta-analysis showed that intravenous acetaminophen was associated with a decreased of VAS scores and opioid consumption (207 mg vs 252 mg, *P* < .05). Additionally, there was a lower risk of nausea and vomiting. The evidence quality for each main outcome was low. Thus, we considered that the overall evidence quality was low. Further research is likely to significantly change confidence in the effect estimate and to change the estimate.

With the widely application of TJA, postoperative pain management is an important clinical challenge. Effective pain control was associated with early mobilization, decreased length of stay, and less postoperative complications. Currently, multimodal pain management was considered to be the optimal choice for pain control after TJA.^[16,17]^ Golladay et al^[18]^ reported that multimodal analgesia were recommended as a part of a pre-emptive approach to pain control in patients undergoing knee or hip arthroplasty.

LIA has been shown to be effective in reducing pain following joint arthroplasty surgeries. Tran and Schwarzkopf^[19]^ reported the use of LIA in management of postoperative TKA pain has been shown to decrease the length of hospital stay and total morphine consumption. Jimenez-Almonte et al^[20]^ conducted a systematic review and showed there were no differences between local infiltration analgesia and peripheral nerve blocks in terms of analgesia or opioid consumption 24 hours after THA. This approach has been criticized because of its short-term pain relief.

Published articles have studied the impact of multimodal analgesia on TJA. Recently, intravenous acetaminophen as an adjunct to multimodal pain management have been shown to improve pain relief, decrease total perioperative morphine consumption, and facilitate early mobilization.^[21,22]^ Acetaminophen could control the endogenous cannabinoid system and inhibit the reuptake of the bioactive mediators by neurons, making it possible to decrease pain. Currently, whether intravenous acetaminophen as an adjunct to multimodal pain management could further reduce opioid consumption was seldom reported and remained controversial. The present meta-analysis showed that intravenous acetaminophen could significantly reduce postoperative pain scores following TJA.

Opioid consumption is also an important indicator for assessing the analgesic effect of acetaminophen. It was normally used as adjunct to a multimodal analgesia protocol. Also the analgesic effect of the additional opioids provides a long postoperative period without any pain experienced by the participants. Lee et al^[23]^ reported there was improvement in pain scores, at rest and on movement, as well as a reduction in incidence of severe pain, in patients who receive PCA analgesia. However, previous studies have frequently reported that patients have experienced drug-related side effects, such as gastrointestinal events, headache, and constipation.^[24,25]^ Effective analgesia protocol is crucial to reduce the consumption of opioids. A substantial number of literatures have demonstrated the intravenous acetaminophen could decrease inpatient narcotic requirements in major orthopedic surgery. However, intravenous acetaminophen for pain control following TJA was seldom reported. Total morphine consumption was 207 mg in experimental groups compared with 252 in the control groups (*P* < .05). The results of meta-analysis showed intravenous acetaminophen was associated with a further reduction of opioid consumption.

Postoperative complications were major concerns following additional opioids. Gastrointestinal events are well-known side effects that are related to the systemic use of morphine. Adequate analgesia protocol could decrease opioid consumption and subsequently decrease the risk of postoperative complications. The present meta-analysis demonstrated that intravenous acetaminophen could significantly decrease the incidence of gastrointestinal events. Large sample sizes of high quality studies should be conducted in the future because only 4 articles were included in the study.

The limitations of our findings are only 4 studies were selected, small sample sizes might affect the overall results. Range of motion was a crucial outcome and we did not perform an analysis due to the limitation of available data. Risk of bias among articles should be considered which may influence our result. Short-term follow-up may cause the underestimation of side effects. Clinical heterogeneity could not be eliminated completely, more RCTs were needed for subgroup analysis.

## 5 Conclusion

Intravenous acetaminophen was efficacious for reducing postoperative pain and opioid consumption than the placebo following total joint arthroplasty. Due to the limited quality of the evidence currently available, more RCTs are needed.
